# The efficacy of analgesics in controlling orthodontic pain: a systematic review and meta-analysis

**DOI:** 10.1186/s12903-020-01245-w

**Published:** 2020-09-18

**Authors:** Caiqi Cheng, Tian Xie, Jun Wang

**Affiliations:** 1 State Key Laboratory of Oral Diseases, Department of Orthodontics, West China College of Stomatology, Sichuan University, No. 14, 3rd Section, Renmin South Road, Chengdu, 610041 China; 2 Department of Stomatology, the First Affiliated Hospital of Shenzhen University, The Shenzhen Second People’s Hospital, Shenzhen, China

**Keywords:** Pain, Orthodontics, Analgesics, Fixed orthodontic appliance, Meta-analysis

## Abstract

**Background:**

Patients who had gone through orthodontic treatment experienced pain and discomfort which could be the highest-ranking reason for treatment disturbance or early termination. Thus, this review aimed to assess the efficacy of analgesics on the relief of pain in orthodontic treatment.

**Methods:**

A computerized literature search was conducted in the databases of EMBASE (via OVID, 1974 to 2019 Week 50), MEDLINE (via OVID, 1946 to Dec 2019), the Cochrane Central Register of Controlled Trials (CENTRAL) (December 2019). The Cochrane Collaboration’s Review Manager 5.3 software was applied in the present study. And methodological quality was evaluated by the Cochrane Risk of Bias Tool.

**Results:**

We identified twelve publications including 587 patients in 19 randomized controlled trials. The results showed that the mean difference of naproxen in visual analogue scale (VAS) were − 1.45 (95% CI -2.72, − 0.19; *P* = .02), − 2.11 (95% CI -3.96, − 0.26; *P* = .03) and − 1.90 (95% CI -3.33, − 0.47; *P* = .009) in 2 h, 6 h and 24 h respectively. As for ibuprofen, the standard mean differences were − 1.10 (95% CI -1.49, − 0.71), − 1.63(95% CI -2.32, − 0.95) and − 1.34 (95% CI -2.12, − 0.55) at 2 h, 6 h, and 24 h, with the overall *P* values all < 0.001. The mean difference of acetaminophen is − 0.68, − 1.34, − 1.91 at three time points and the overall *P* values all < 0.01.

**Conclusions:**

This meta-analysis suggests that the use of analgesics is effective for patients in controlling orthodontic pain. Ibuprofen and naproxen are both of stable analgesic effects which could peak at 6 h, while the analgesic effect of acetaminophen increases steadily from 2 h through 24 h. Compared with ibuprofen and acetaminophen, naproxen shows a stronger analgesic effect either at 2 h or 6 h, and its effect lasts to 24 h.

## Background

Despite all the technological advances in orthodontics, pain is inevitable and hard to bear in the process of orthodontic treatment. The existing literature suggests that all the clinical operation (initial archwire placement, separator placement and activations) can be the root cause of orthodontic pain. There is no denying the fact that fixed appliances could produce more pain compared with functional or removable appliances. A survey of patients who had ever gone through orthodontic treatment found that 91% experienced pain during treatment and 50% had difficulty in eating and were limited even in their daily life [1, 2]. Similarly, pain has been reported to be the highest-ranking reason for treatment disturbance or early termination [3, 4]. However, not enough attentions have been paid to the orthodontic pain neither in clinic or research, and there is no universal recommendation on the interventions for pain relief as well. Ways to relieve orthodontic pain have differed greatly among which nonsteroidal anti-inflammatory drugs (NSAIDs) are the most popular drugs for pain controlling. In specific, ibuprofen and paracetamol/acetaminophen are drugs commonly recommended for this purpose, whereas other methods such as low-level laser therapy, anesthetic gel, transcutaneous electrical nerve stimulation and even bite wafers, have also been mentioned in some trials. Orthodontic tooth movement leads to inflammation of the periodontal membrane and the dental pulp response, which stimulates the release of a variety of biochemical mediators bringing about pain sensation [5, 6]. NSAIDs function by blocking the synthesis of arachidonic acid in the prostaglandin production cycle reducing the formation of prostaglandins. As we all know that prostaglandin is the main mediators in the process of inflammatory reaction after the orthodontic force [7, 8]. Nevertheless, there is lack of adequate clinical evidence to assess the validity of various analgesics, let alone a standard medication protocols up to date.

The purpose of this systematic review and meta-analysis is to compare the efficacy of commonly used analgesics in orthodontic pain management and develop a recommendation on the medication for pain management.

## Materials and methods

### Search strategy

We searched online databases, including EMBASE (via OVID, 1974 to 2019 Week 50), MEDLINE (via OVID, 1946 to Dec 2019) and the Cochrane Central Register of Controlled Trials (CENTRAL) (December 2019). World Health Organization International Clinical Trials Registry Platform was searched for clinical trials in progress. No restrictions about language, country or date of publication. MeSH terms and free text words used for orthodontic pain were combined as follows: “orthodontics” or “orthodontic treatment” or “tooth movement”, “pain” or “discomfort”, “analgesia” or “NSAIDS” or “ibuprofen” or “acetaminophen” with every possible combination considered. Reference lists of all the studies included were checked. Efforts were made to contact all corresponding authors for more information when data were missing.

### Data extractions

No restrictions were imposed to maintain more specific methodological characteristics on the search. The inclusion and exclusion criteria are listed in Table 1. Two independent investigators (C.-Q. C. and T.X.) selected studies based on titles and abstracts. The names of the journals or authors were hidden in the filter and articles accord with the criteria above were included. Disagreements regarding the inclusion were resolved by discussion to reach consensus in this review. Then full texts were evaluated in the same way by the reviewers independently. The data were collected by two authors (C.-Q. C. and T.X) through consultation on the items including first author, publication year, original country, case number, type of design, medication dose and outcomes. Attempts had been made to contact the authors of the selected trials by e-mail for relevant data that were not specified in the articles.

**Table 1:** Inclusion and exclusion criteria

**Inclusion Criteria**	
1. The study was a randomized controlled trial (RCT);	
2. studies compared NSAIDS with placebo for orthodontic pain using quantitative outcome data;	
3. As for the experiment intervention, Participators were not allowed to be currently taking any antibiotics or analgesics, with no teeth extractions at least two weeks before the appointment and on contraindications or adverse reactions to NSAIDS;	
4. The outcomes of pain perception were measured by either visual analog scale (VAS) or a questionnaire for pain perception;	
5. Duration of follow-up was assessed and defined as short term (eg: 2 h, 6 h, 24 h,7 days).	
**Exclusion Criteria**	
1. Studies were cohort studies, review articles, case reports, descriptive studies, opinion articles, and abstracts;	
2. The subjects had systemic disease or chronic pain or histories of neurologic and psychiatric disorders;	
3. Patients had any acute or chronic dental, periodontal or gingival problems which could cause pain.	

### Risk of bias assessment

The methodological quality of studies was assessed using the Cochrane Risk of Bias 2 (RoB-2) [9]. The tool assesses five areas of potential bias including: (1) randomization process, (2) deviations from the intended interventions, (3) missing outcome data, (4) measurement of the outcome, and (5) selection of the reported result. Each domain assessed and each study overall is shown to have either a low risk of bias, some concerns relating to the risk of bias, or a high risk of bias, as determined by a validated a priori algorithm. Two reviewers independently read the articles to assess the trials more accurately.

### Data synthesis and analysis

Review Manager 5.3 from The Cochrane Collaboration was implemented in this review. The DerSimonian and Laird random effects model was used to identify the VAS outcome of orthodontic pain. The estimates were represented as mean difference (MD) as well as 95% confidence interval (CI). Heterogeneity was identified using Cochran’s Q test and quantified by the I^2^statistic and Tau2 as measurements of inconsistent level and between-study variance [10]. Sensitivity analysis integrating RoB-2 assessment was conducted to test the stability of the results. We also conducted subgroup analysis according to orthodontic treatment pattern (separator placement or archwire placement). To explore possible publication bias, a funnel plot would be performed when the number of studies pooled was over 10.

## Results

### Search results

**Fig. 1:**
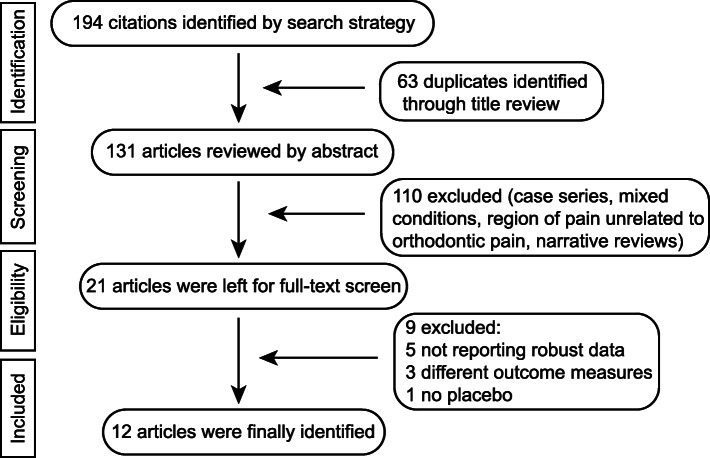
Flow chart of the selection process of related publications

194 publications were collected after the first screening (last updated on Dec. 2019). Twenty-one studies remained for full-text screen and 12 studies were finally identified in the present review as shown in the flow chart (Fig. 1). In total, 12 eligible trials which were all randomized placebo-controlled trials comprising 587 subjects which met the inclusion criteria. The characteristics and data summary of these studies are displayed in Table 2 [11–22].

**Table 2:** Characteristics of the included studies

Study	Country	Design	Age (years)	Sex (% female)	Orthodontic treatment	Interventions	Evaluation intervals	Outcome measures
Bernhardt 2001 [11]	America	RCT	Mean12	51%	separator placement	Ibuprofen(400 mg),placebo	2 h, 6 h, at night, 24 h, 2 d, 3 d, 7 d	VAS
Farzanegan 2012 [12]	Iran	RCT	13–18	100%	archwire placement	Ibuprofen(400 mg),placebo, chewing gum,soft viscoelastic wafer, and hard viscoelastic wafer	2 h, 6 h, at night, 24 h, 2 d, 3 d, 7 d	VAS
Kohli 2011 [13]	India	RCT	13–20	50%	separator placement	Ibuprofen(400 mg), placebo	2 h, 6 h, at night, 24 h, 2 d, 3 d, 7 d	VAS
Minor 2009 [14]	America	RCT	13–30	25%	separator placement	Ibuprofen(400 mg),placebo	2 h, 6 h, bedtime,awakening,24 h	VAS
Patel 2011 [15]	America	RCT	18–30	46%	separator placement	Ibuprofen, naproxen sodium, acetaminophen, placebo (OTC)	2 h, 6 h, bedtime,awakening,24 h	VAS
Polat 2005 [16]	Turkey	RCT	10–24	38%	archwire placement	Ibuprofen(400 mg),placebo, naproxen sodium	2 h, 6 h, at night, 24 h, 2 d, 3 d, 7 d	VAS
Salmassian 2009 [17]	America	RCT	12–18	48%	separator placement	Ibuprofen(400 mg),acetaminophen(600 mg),placebo	0 h,3 h, 7 h, 19 h,24 h, 31 h,48 h, 3 d,4d, 7 d	VAS
Sudhakar 2014 [18]	India	RCT	14–21	50%	separator placement	Ibuprofen(400 mg),acetaminophen(650 mg),aspirin(300 mg),placebo	2 h, 6 h, bedtime,24 h,2 d, 3 d, 7 d	VAS
Gupta 2014 [19]	India	RCT	15–22	49%	archwire placement	Acetaminophen(500 mg), etoricoxib(60 mg), placebo	2 h, 6 h, at night, 24 h,2d,3d	VAS
Eslamian 2017 [20]	Iran	RCT	14–20	68%	separator placement	Naproxen, placebo	2 h, 6 h, 24 h,2d,3d,7d	VAS
Nik 2016 [21]	Iran	RCT	Mean15	56%	separator placement	Acetaminophen(650 mg), ibuprofen(400 mg), and placebo	0 h, 2 h, 6 h, bedtime, 24 h	VAS
Kaur 2019 [22]	India	RCT	Mean15	70%	separator placement	Acetaminophen(500 mg), verbal behavior modification, placebo	6 h, 24 h,2d,3d,4d,5d,6d,7d	VAS

### Study quality

**Fig. 2:**
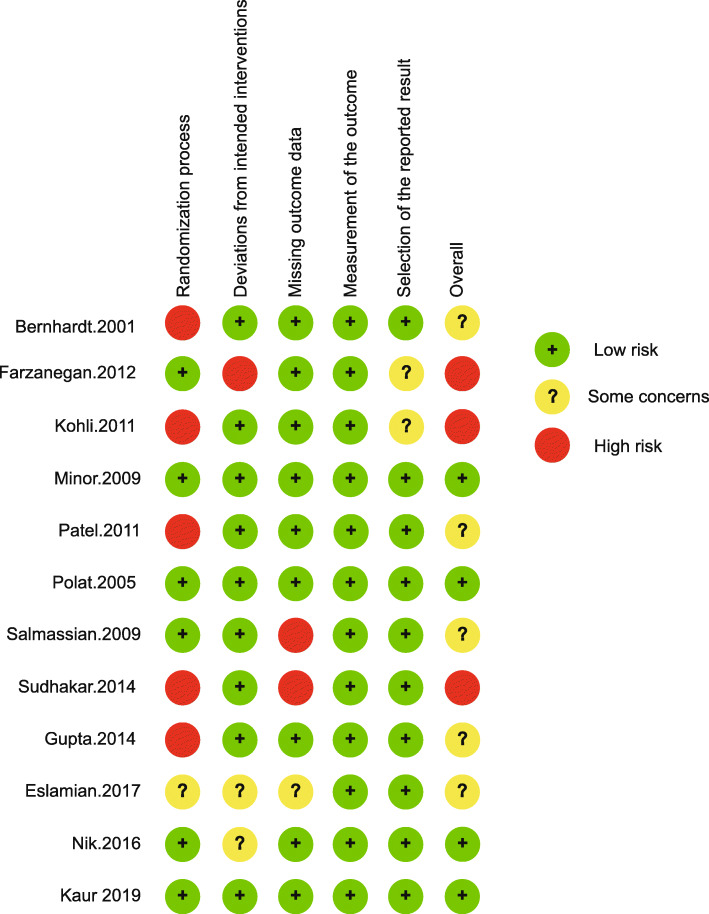
Risk of bias summary: review authors’ judgments of each risk of bias item for each included study according to RoB-2

As evaluated by the RoB-2, 5 of 12 studies showed some concerns across all domains and three studies exhibited high risks of potential bias. In specific, the randomization process domain demonstrated several high risks of bias and the measurement of the outcome were all low risks of bias. Two studies suggested high risk of bias as a result of missing outcome data, while one study showed some concerns. These issues related to studies where more than 5% of participants were lost to follow-up or had missing data, yet analyses to assess those lost to follow-up were not conducted. Deviations from the intended intervention also suggested a need for caution, with one study showing high risk and two some concerns. Risk of bias due to selection of the reported result revealed two studies as some concerns (Fig. 2).

Sensitivity analysis was performed and the results were consistent after removing trials with high risks of bias as shown in Fig. S1, S2 and S3. Additionally, the subgroup analysis of separator placement did not reduce the heterogeneity significantly but demonstrated the consistent results with the total analysis (Fig. S4, S5 and S6).

### Ibuprofen vs placebo group

**Fig. 3:**
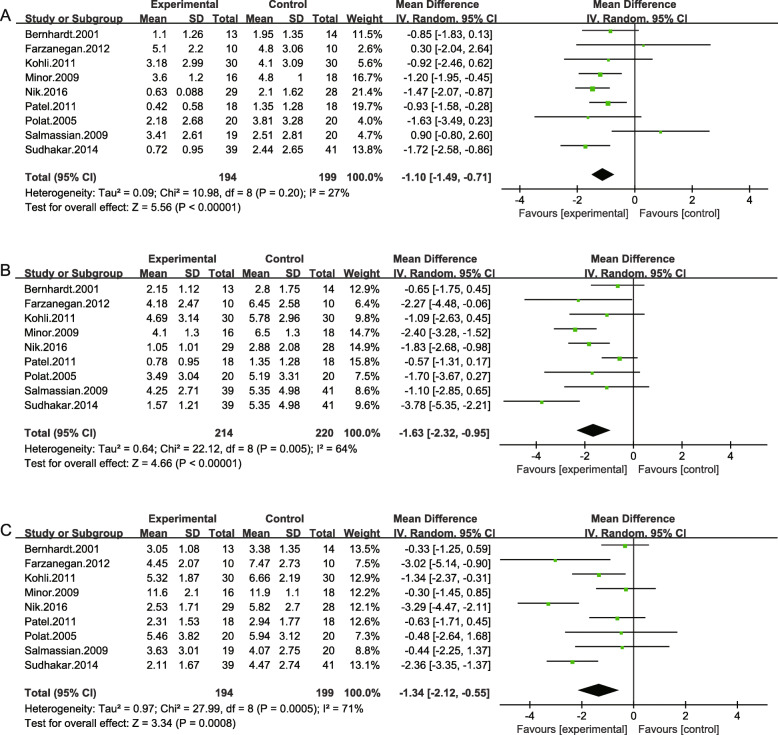
Results of the meta-analysis. Pooled estimate of VAS scores of ibuprofen vs. placebo at 2 h(**a**), 6 h(**b**) and at 24 h(**c**) respectively after orthodontic treatment. The effect of pain relief is depicted as MD and its 95% CI. I ^2^ represents the amount of heterogeneity

Nine of the twelve included studies measured the same outcome (ibuprofen vs placebo). The meta-analysis of nine studies involving 393 patients showed that ibuprofen was more effective in controlling orthodontic pain compared with placebo at different time points. The standard mean differences were − 1.10 (95% CI -1.49, − 0.71), − 1.63 (95% CI -2.32, − 0.95) and − 1.34 (95% CI -2.12, − 0.55) at 2 h, 6 h and 24 h respectively, with the overall *P* values all < 0.001, indicating that the results favored the ibuprofen group more than the placebo group (Fig. 3).

### Acetaminophen vs placebo group

**Fig. 4:**
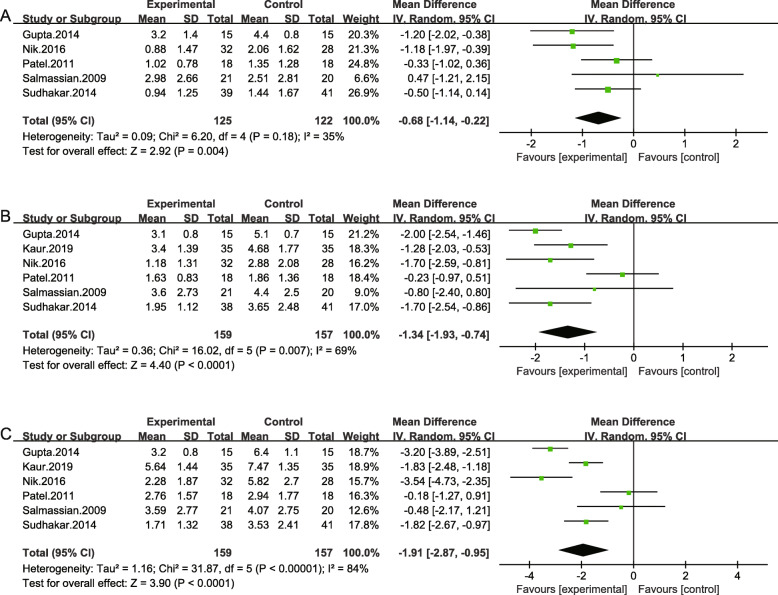
Results of the meta-analysis. Pooled estimate of VAS scores of acetaminophen vs. placebo at 2 h(**a**), 6 h(**b**) and at 24 h(**c**) respectively after orthodontic treatment. The effect of pain relief is depicted as MD and its 95% CI. I ^2^ represents the amount of heterogeneity

Six investigators reported the efficacy between acetaminophen and placebo including 316 patients at different time points. The standard mean differences of 2 h were − 0.68 (95% CI -1.14, − 0.22; *P* = .004) and the heterogeneity was acceptable (χ^2^ = 6.20, *P* = 0.18, I^2^ = 35%). At 6 h and 24 h, the mean differences were − 1.34 (95% CI -1.93, − 0.74; *P* < .0001) and − 1.91 (95% CI -2.87, − 0.95; P < .0001). The results of Patel et al. and Salmassian et al. indicated no statistical difference for pain relief at the all three time points (Fig. 4).

### Naproxen vs placebo group

**Fig. 5:**
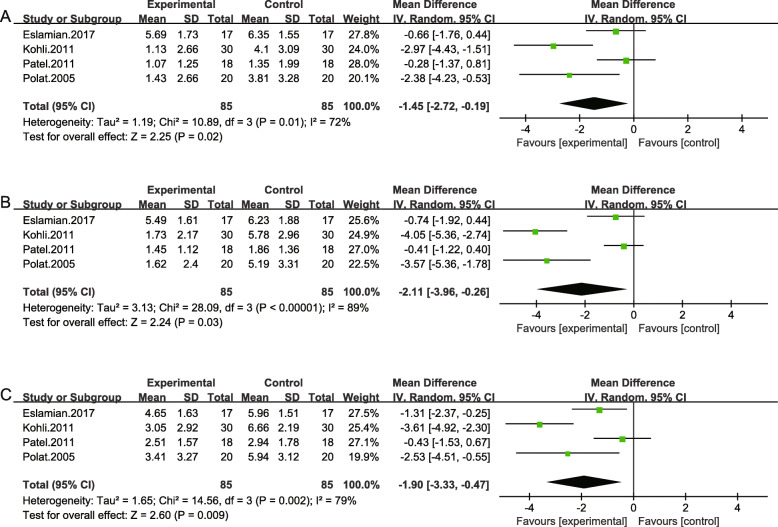
Results of the meta-analysis. Pooled estimate of VAS scores of naproxen vs. placebo at 2 h(**a**), 6 h(**b**) and at 24 h(**c**) respectively after orthodontic treatment. The effect of pain relief is depicted as MD and its 95% CI. I ^2^ represents the amount of heterogeneity

Four studies totally 170 patients compared naproxen with placebo, in which two studies (Kohli et al. and Polat et al.) suggested significant pain relief in naproxen group. While Patel et al. and Eslamian et al. showed no statistical difference between the naproxen and the placebo. The mean differences of three time points (2 h, 6 h, 24 h) were − 1.45 (95% CI -2.72, − 0.19; *P* = .02), − 2.11 (95% CI -3.96, − 0.26; *P* = .03) and − 1.90 (95% CI -3.33, − 0.47; *P* = .009) respectively (Fig. 5).

## Discussion

Orthodontic treatment has widely been accepted by the public, but it is still associated with pain regardless of the technique advance in orthodontics. Moderate statistical evidence has been shown for efficacy of analgesics managing orthodontic pain in the short term. An average decrease in VAS of 15 mm was recorded, which could be significant in clinic [23, 24]. From our analysis, the use of NSAIDs had statistically significant analgesic effects for patients in controlling orthodontic pain among which naproxen shows a stronger analgesic effect either at 2 h or 6 h, and its effect lasts to 24 h compared with ibuprofen and acetaminophen. Ibuprofen and naproxen are both of stable analgesic effect which could peak at 6 h, while the analgesic effect of acetaminophen increases steadily from 2 h through 24 h. The trials of Negan et al. revealed that patients may feel painful within 4 h after the first archwire placement or separator placement. And pain peaked at 24 h and gradually disappeared in a week [25]. The use of analgesics may help patients get through the hard times after orthodontic appointments.

The present study has included larger quantities of articles than previous reviews, also the meta-analysis were more comprehensive and convictive. Of twelve included trials, nine reported using separator placement and three archwire placement. It is believed that these appliances resulted in similar pain experience, and therefore their data were synthesized in this meta-analysis. Dose assessment was important for the analysis of analgesics research, which could recommend an applicable dose and avoid unnecessary effects. Our findings showed that the mean dosage of ibuprofen (400 mg) and acetaminophen (600 mg) one hour before the treatment and six hours after the treatment are optimum. As for naproxen, it exhibits a long half-life of 50–60 h, which permits once daily dosing. The recommended dosage of naproxen is 20–30 mg once daily. We recommend a multi-center, pragmatic trial in an appropriately powered study to test the effectiveness of parameters of this order.

The concern about NSAIDs is that they may delay the rate of tooth movement [26–28]. Walker et al. reported that NSAIDs inhibit the cyclooxygenase pathway and therefore the production of PGE, and it was thought that NSAIDs may inhibit the osteoclastic activity necessary for tooth movement and slow the speed of tooth movement [29]. Acetaminophen is preferred on grounds that it is inactive as an anti-inflammatory agent in peripheral tissues and does not prevent prostaglandin synthesis, which means that it has no influence on the speed of tooth movement [6, 30]. In fact, the dosage applied in clinic is relatively low and the time is short. In a healthy patient, the dosage of these analgesics would be eliminated from the body before the tooth movement starts [31]. Therefore, administrations of low doses analgesics for a short period will not prevent the potential of slowing tooth movement process. Researchers also pay attention to some long-acting NSAIDs (piroxicam and tenoxicam) and cyclooxygenase-2 (COX-2) inhibitors (valdecoxib) these days. Unfortunately, we could not make meta-analysis because of the limited amount of relevant evidence. It is expected for more well work-out studies to address the efficacy of these analgesics.

Several limitations in the present study should be acknowledged. Firstly, the gender distributions between subgroups were not evenly distributed in two of twelve included studies [11, 17]. Although large numbers of studies have found no difference between the genders after orthodontic treatment [32, 33], two studies found that girls reported more pain and ulcerations than boys [2, 34]. Secondly, some studies showed a relative high heterogeneity but we did not find key covariates that was responsible for heterogeneity. We carried out sensitive analysis restricting to same orthodontic intervention in order to determine whether different orthodontic treatment was the cause. The results showed that the heterogeneity still remained although it did account for some heterogeneity, indicating that other unreported confounding factors might affect the heterogeneity. Additionally, it was reported that peak pain intensity may vary between the archwire placement and separator placement [35], the results of sensitive analysis were comparable before and after excluding 3 studies using archwire placement in the present study. Also, we have to admit that we did not pre-register this study, but our analysis was conducted in strict accordance with the systematic review process. Furthermore, a clinical medication should take all factors which affect perception of pain into consideration including the age, the level of anxiety and self-medication history so as to decrease potential risks that can lead to severe complications.

## Conclusions

Based on the data available, the use of analgesics is effective for patients in controlling orthodontic pain. Ibuprofen and naproxen are both of stable analgesic effect which could peak at 6 h, while the analgesic effect of acetaminophen increases steadily from 2 h through 24 h. And naproxen shows a stronger analgesic effect either at 2 h or 6 h, and its effect lasts to 24 h compared with ibuprofen and acetaminophen. More well-designed RCTs about long-acting NSAIDs and COX-2 inhibitors will be needed to draw a comprehensive conclusion.

## Supplementary information

**Additional file 1: Figure S1.** Sensitivity analysis excluding trials at high risk of bias. Pooled estimate of VAS scores of ibuprofen vs. placebo at 2 h(A), 6 h(B) and at 24 h(C) respectively after removing studies with high risk of bias. The effect of pain relief is depicted as MD and its 95% CI. I ^2^ represents the amount of heterogeneity.

**Additional file 2: Figure S2.** Sensitivity analysis excluding trials at high risk of bias. Pooled estimate of VAS scores of acetaminophen vs. placebo at 2 h(A), 6 h(B) and at 24 h(C) respectively after removing studies with high risk of bias. The effect of pain relief is depicted as MD and its 95% CI. I ^2^ represents the amount of heterogeneity.

**Additional file 3: Figure S3.** Sensitivity analysis excluding trials at high risk of bias. Pooled estimate of VAS scores of naproxen vs. placebo at 2 h(A), 6 h(B) and at 24 h(C) respectively after removing studies with high risk of bias. The effect of pain relief is depicted as MD and its 95% CI. I ^2^ represents the amount of heterogeneity.

**Additional file 4: Figure S4.** Subgroup analysis of separator placement. Pooled estimate of VAS scores of ibuprofen vs. placebo at 2 h(A), 6 h(B) and at 24 h(C) respectively in the group of separator placement. The effect of pain relief is depicted as MD and its 95% CI. I ^2^ represents the amount of heterogeneity.

**Additional file 5: Figure S5.** Subgroup analysis of separator placement. Pooled estimate of VAS scores of acetaminophen vs. placebo at 2 h(A), 6 h(B) and at 24 h(C) respectively in the group of separator placement. The effect of pain relief is depicted as MD and its 95% CI. I ^2^ represents the amount of heterogeneity.

**Additional file 6: Figure S6.** Subgroup analysis of separator placement. Pooled estimate of VAS scores of naproxen vs. placebo at 2 h(A), 6 h(B) and at 24 h(C) respectively in the group of separator placement. The effect of pain relief is depicted as MD and its 95% CI. I ^2^ represents the amount of heterogeneity.

## Data Availability

The summary of data extraction in this study is available upon request to the corresponding author.

## Abbreviations

VAS: visual analogue scale
NSAIDs: nonsteroidal anti-inflammatory drugs
RoB-2: the Cochrane Risk of Bias 2
MD: mean difference
CI: confidence interval
COX-2: cyclooxygenase-2
RCT: randomized controlled trial
