# Supplementary figures and images for: The efficacy of analgesics in controlling orthodontic pain: a systematic review and meta-analysis
Source: BMC Oral Health. 2020 Sep 18;20:259. doi: 10.1186/s12903-020-01245-w (PMC7501721; doi:10.1186/s12903-020-01245-w)

A

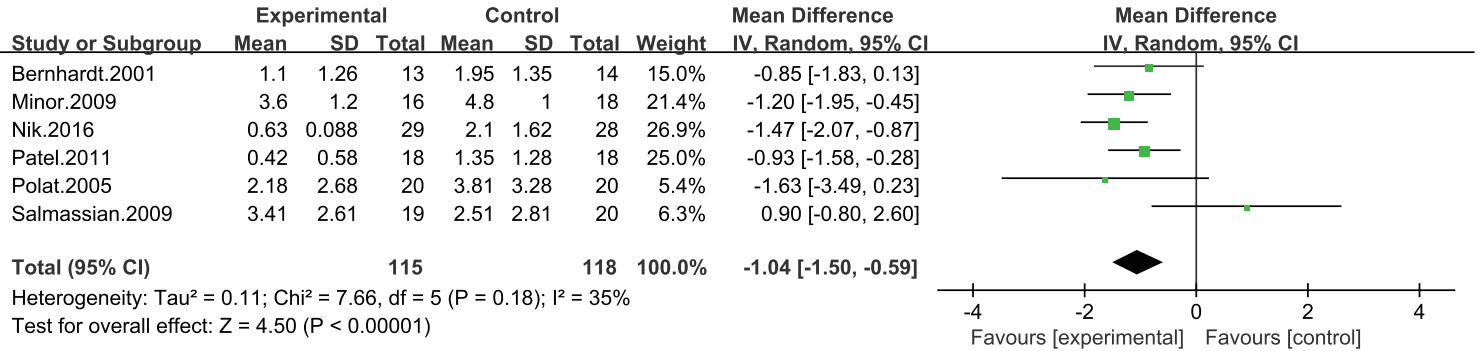

B

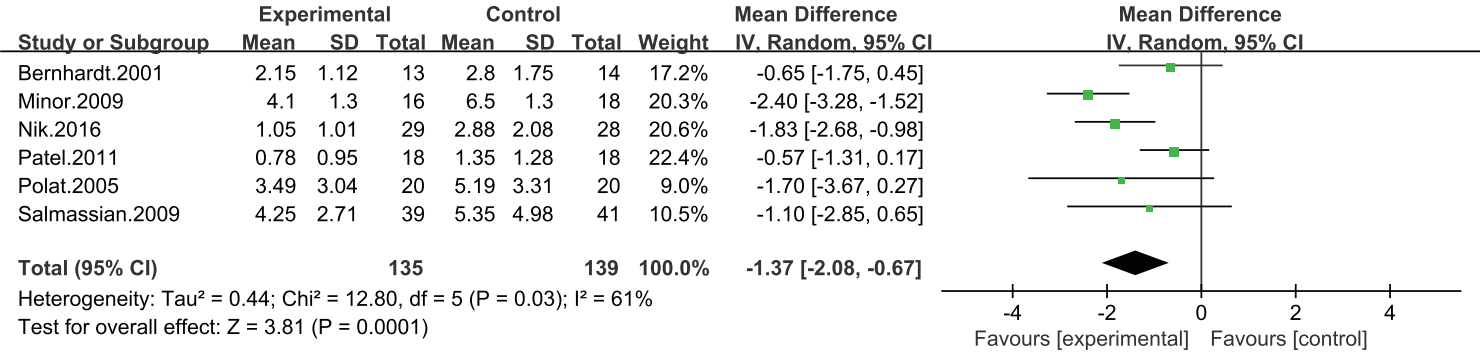

C

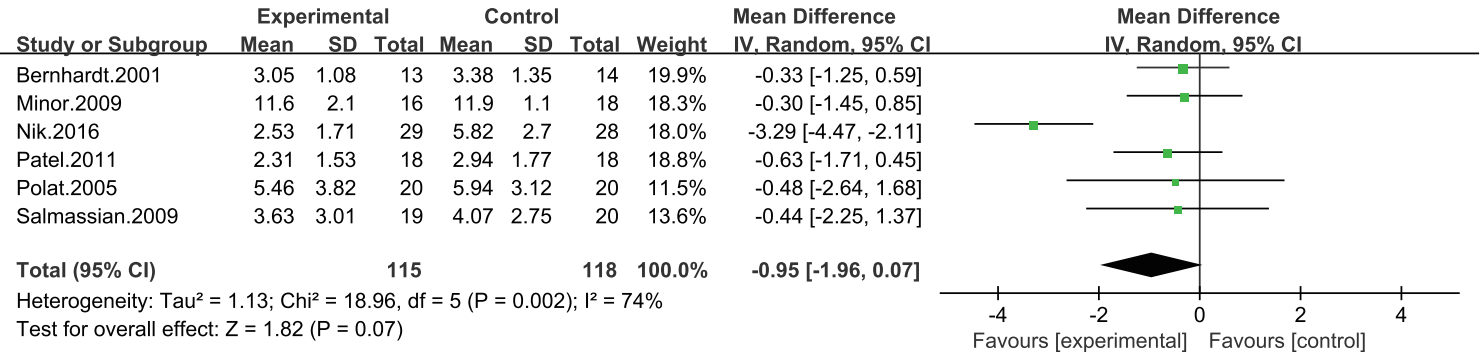

Supplement: Supplementary file 1 — Additional file 1: Figure S1. Sensitivity analysis excluding trials at high risk of bias. Pooled estimate of VAS scores of ibuprofen vs. placebo at 2 h(A), 6 h(B) and at 24 h(C) respectively after removing studies with high risk of bias. The effect of pain relief is depicted as MD and its 95% CI. I 2 represents the amount of heterogeneity. [file 12903_2020_1245_MOESM1_ESM.pdf]

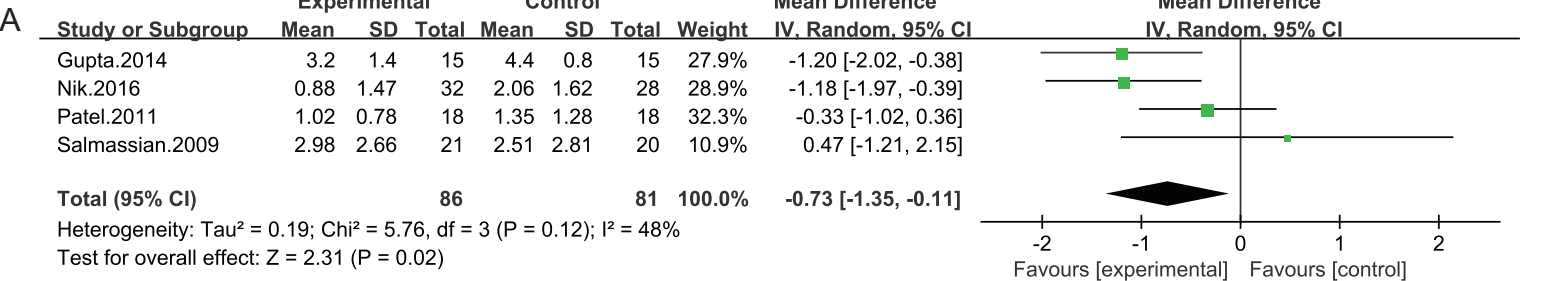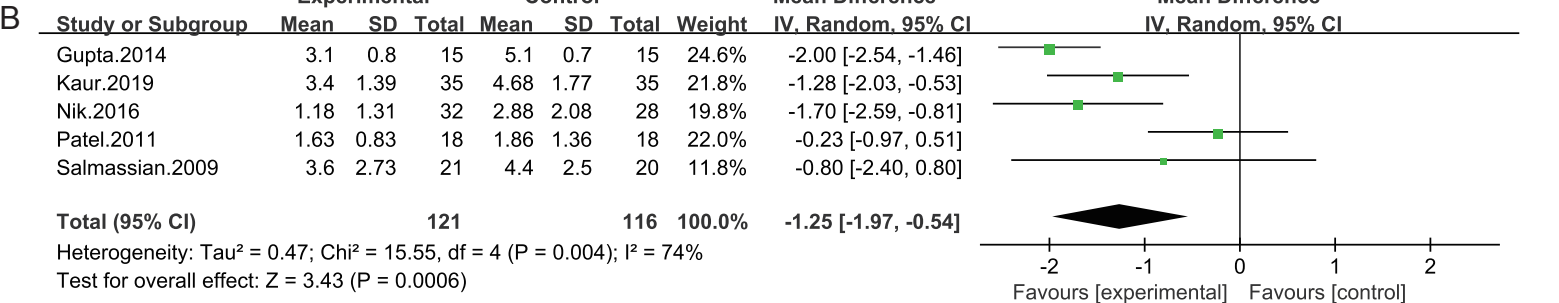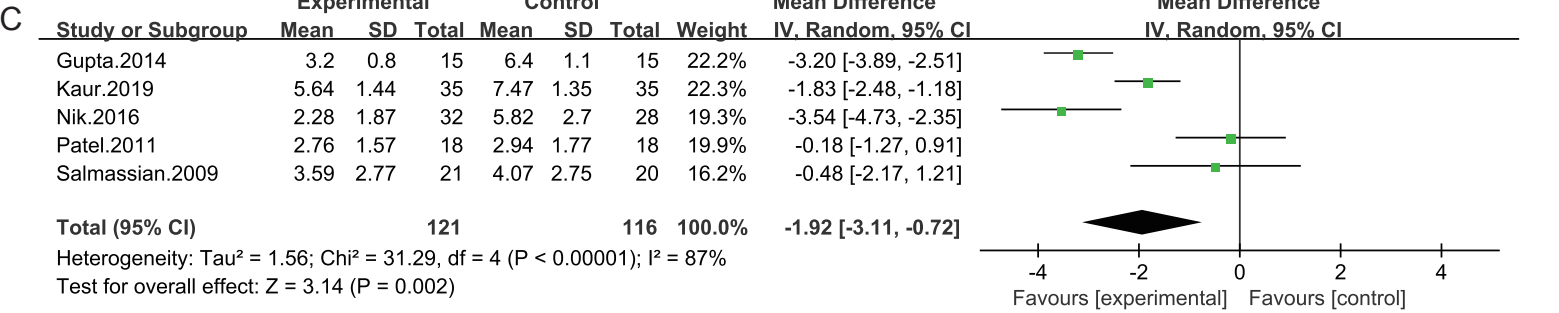

Supplement: Supplementary file 2 — Additional file 2: Figure S2. Sensitivity analysis excluding trials at high risk of bias. Pooled estimate of VAS scores of acetaminophen vs. placebo at 2 h(A), 6 h(B) and at 24 h(C) respectively after removing studies with high risk of bias. The effect of pain relief is depicted as MD and its 95% CI. I 2 represents the amount of heterogeneity. [file 12903_2020_1245_MOESM2_ESM.pdf]

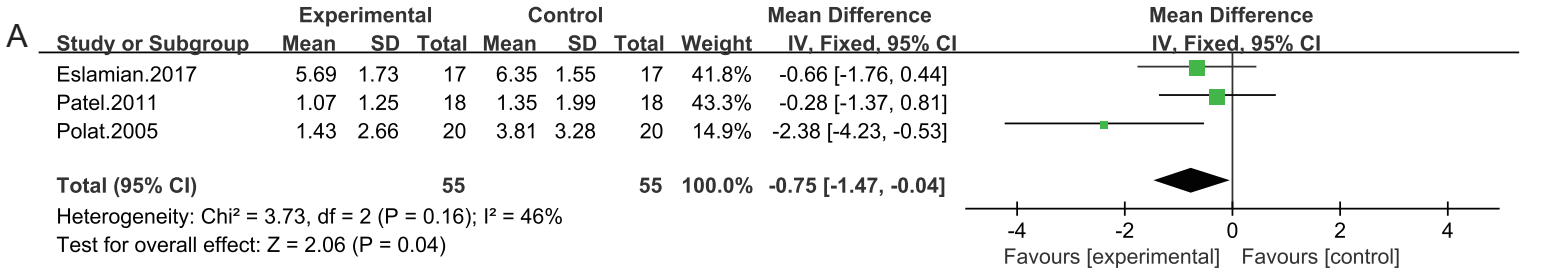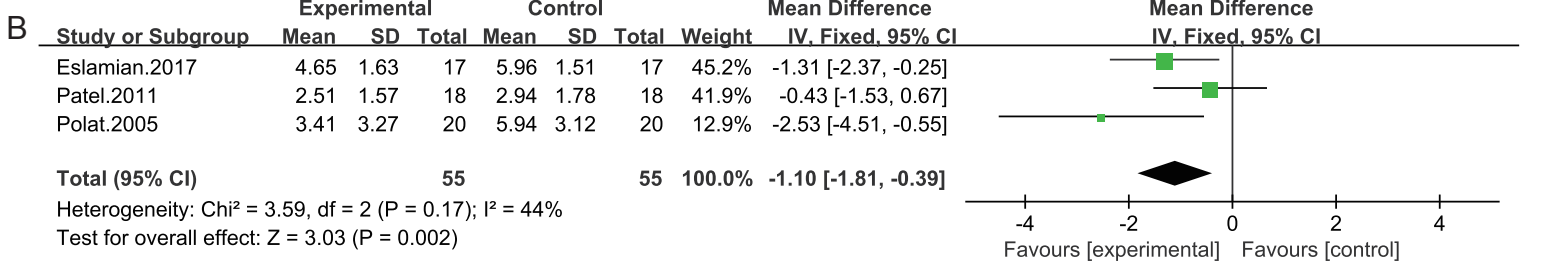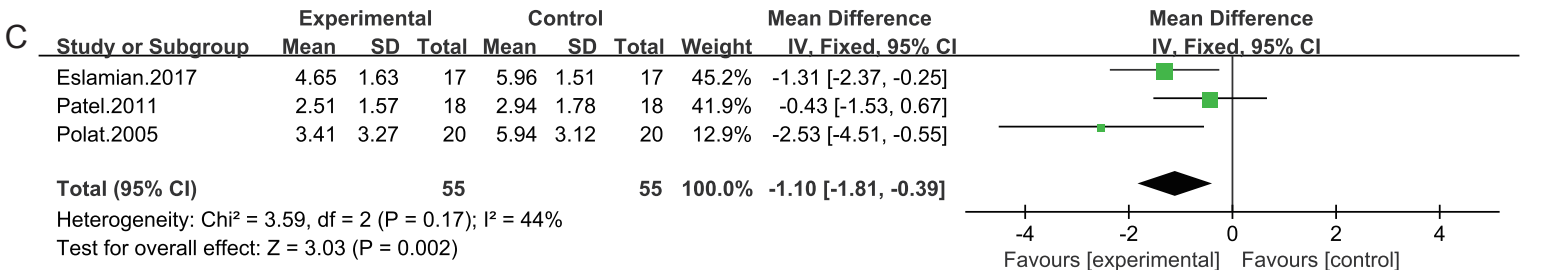

Supplement: Supplementary file 3 — Additional file 3: Figure S3. Sensitivity analysis excluding trials at high risk of bias. Pooled estimate of VAS scores of naproxen vs. placebo at 2 h(A), 6 h(B) and at 24 h(C) respectively after removing studies with high risk of bias. The effect of pain relief is depicted as MD and its 95% CI. I 2 represents the amount of heterogeneity. [file 12903_2020_1245_MOESM3_ESM.pdf]

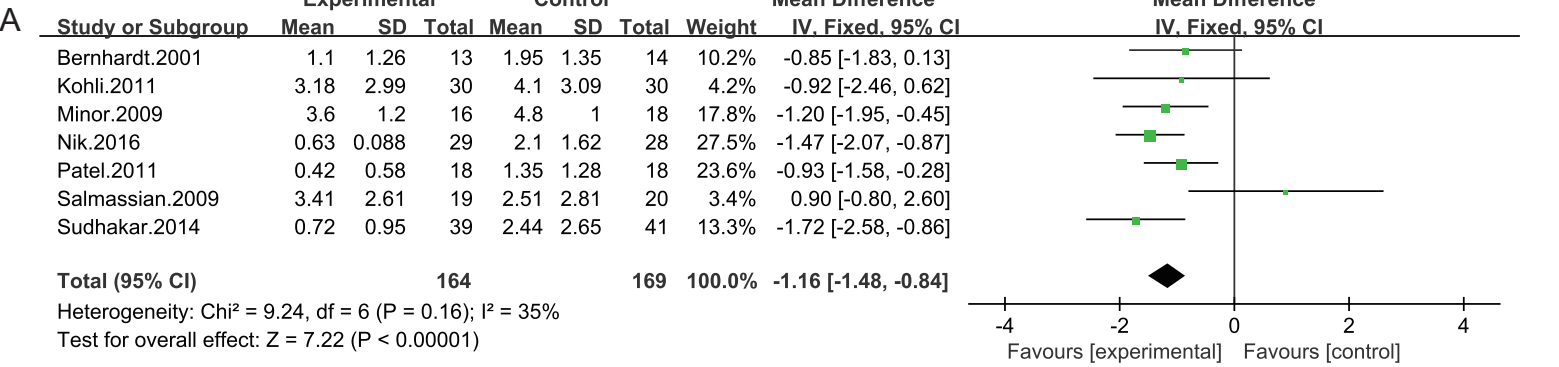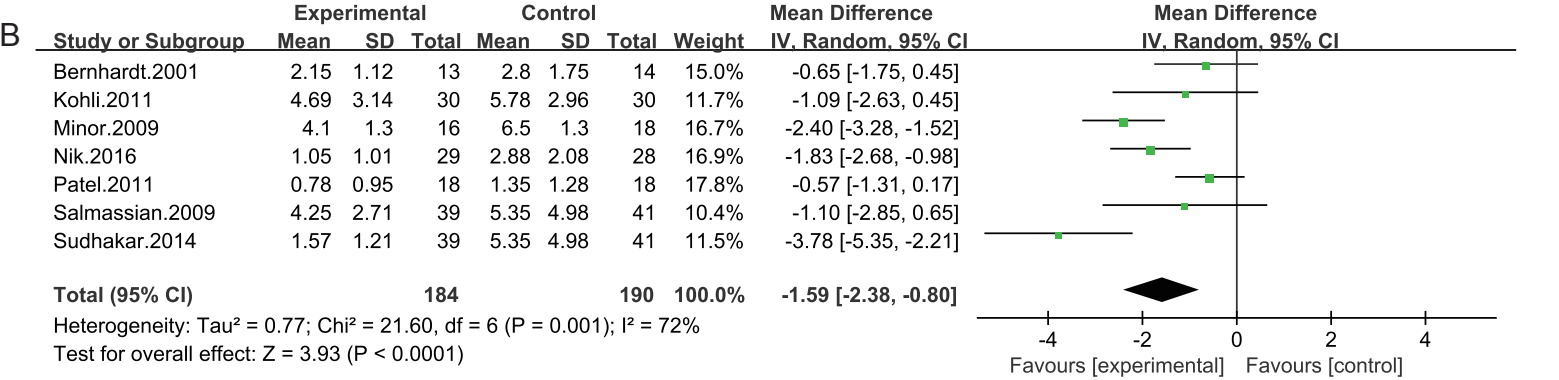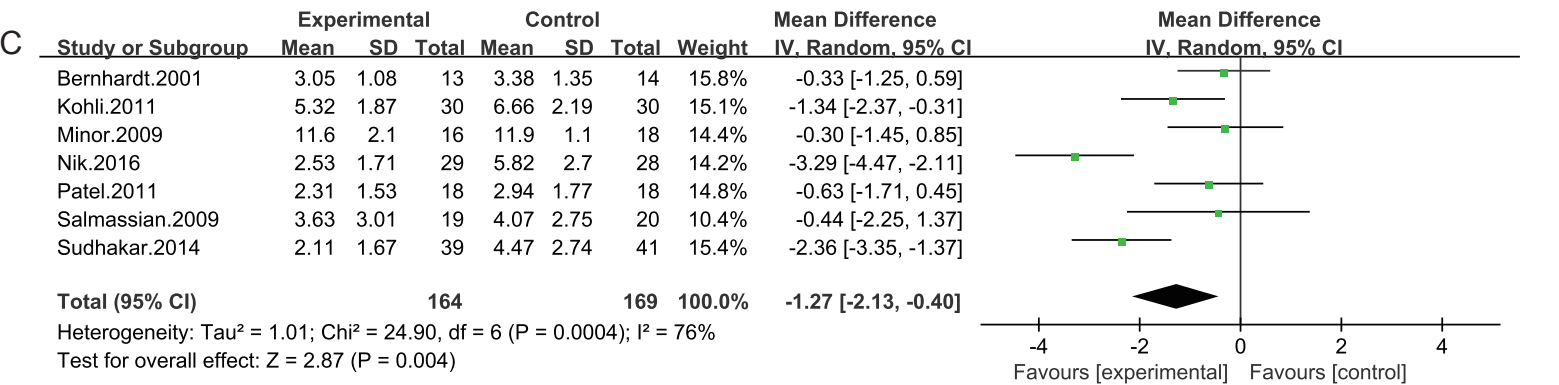

Supplement: Supplementary file 4 — Additional file 4: Figure S4. Subgroup analysis of separator placement. Pooled estimate of VAS scores of ibuprofen vs. placebo at 2 h(A), 6 h(B) and at 24 h(C) respectively in the group of separator placement. The effect of pain relief is depicted as MD and its 95% CI. I 2 represents the amount of heterogeneity. [file 12903_2020_1245_MOESM4_ESM.pdf]

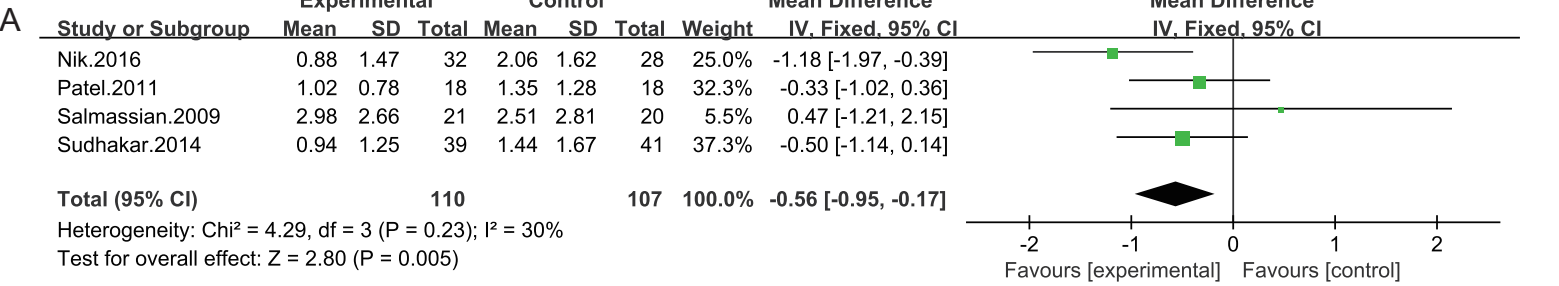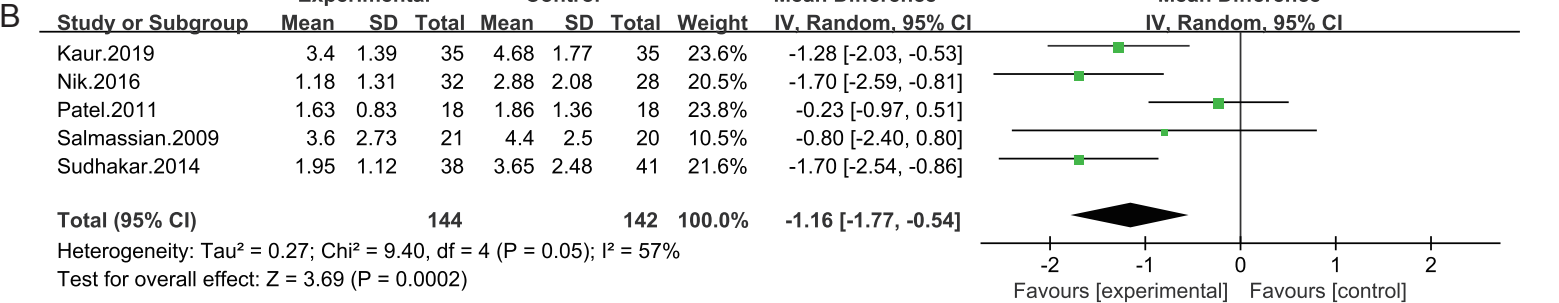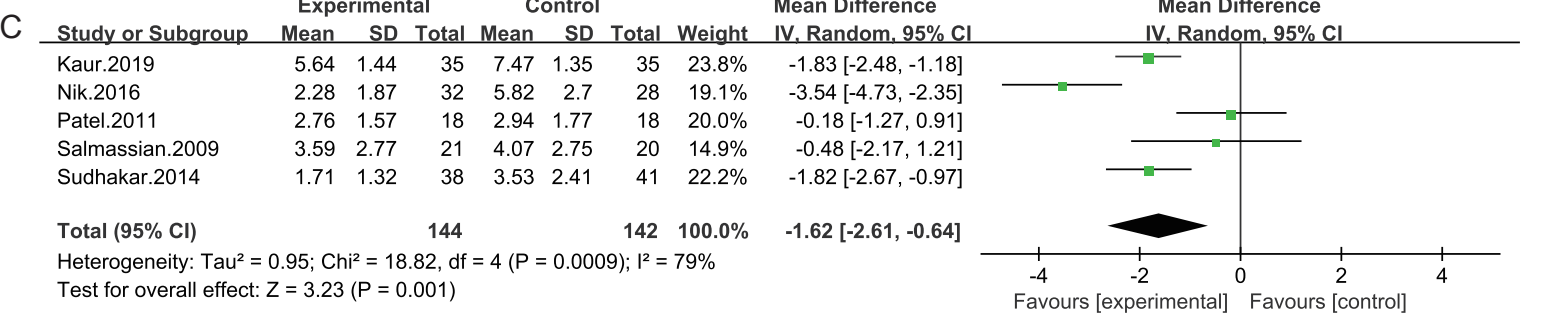

Supplement: Supplementary file 5 — Additional file 5: Figure S5. Subgroup analysis of separator placement. Pooled estimate of VAS scores of acetaminophen vs. placebo at 2 h(A), 6 h(B) and at 24 h(C) respectively in the group of separator placement. The effect of pain relief is depicted as MD and its 95% CI. I 2 represents the amount of heterogeneity. [file 12903_2020_1245_MOESM5_ESM.pdf]

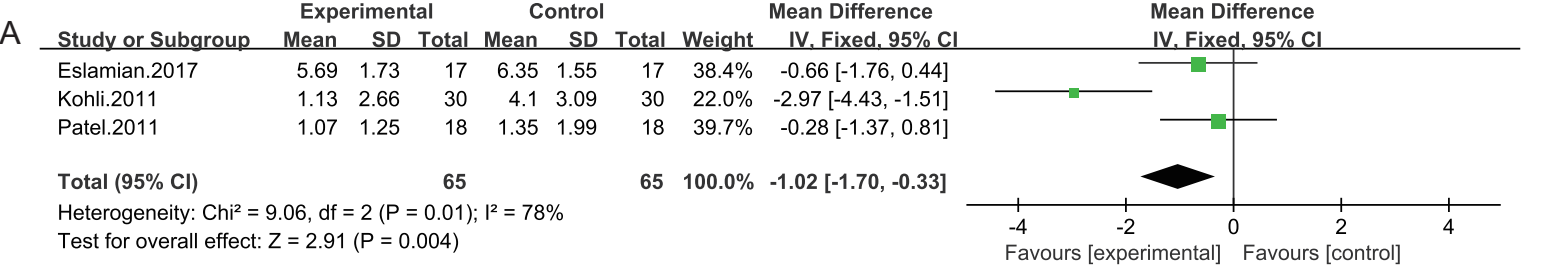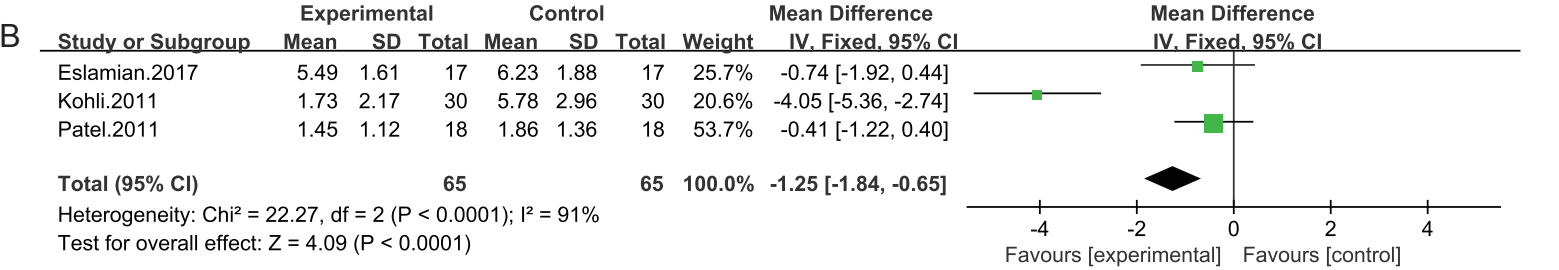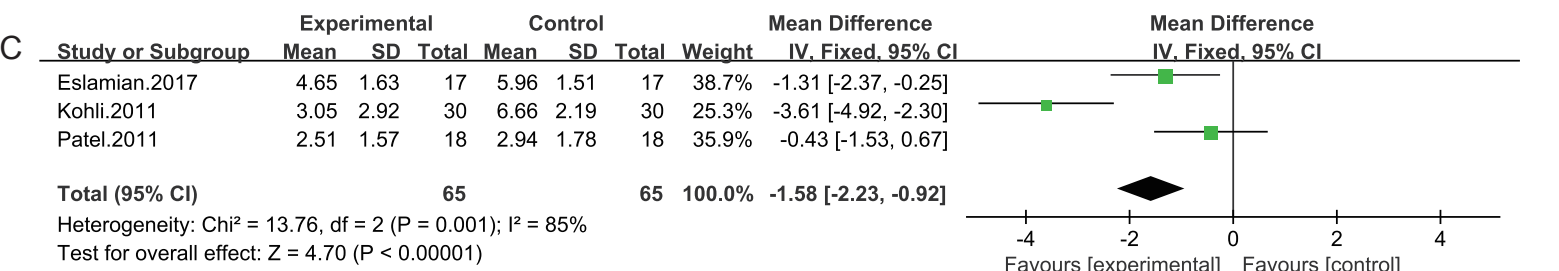

Supplement: Supplementary file 6 — Additional file 6: Figure S6. Subgroup analysis of separator placement. Pooled estimate of VAS scores of naproxen vs. placebo at 2 h(A), 6 h(B) and at 24 h(C) respectively in the group of separator placement. The effect of pain relief is depicted as MD and its 95% CI. I 2 represents the amount of heterogeneity. [file 12903_2020_1245_MOESM6_ESM.pdf]
